# 578. Metformin reduces the risk of Long COVID or Death over 6 months in an Emulated Target Trial of Primarily Omicron-infected Adults without Diabetes or Prediabetes: a New-User, Active-Comparator Analysis Using the National COVID Cohort Collaborative (N3C) Electronic Health Record Database. This research was supported in part by the Intramural/Extramural research program of the National Center for Advancing Translational Science, NIH

**DOI:** 10.1093/ofid/ofae631.016

**Published:** 2025-01-29

**Authors:** Carolyn Bramante, Til Sturmer, Jared Huling, John Buse, Steve Johnson, Christopher Lindsell, Thomas Stewart, David Sahner, Sarah Dunsmore, Eric Topol

**Affiliations:** University of Minnesota, Minneapolis, MN; University of North Carolina, Chapel Hill, Chapel Hill, North Carolina; University of Minnesota, Minneapolis, MN; University of North Carolina, Chapel Hill, Chapel Hill, North Carolina; University of Minnesota, Minneapolis, MN; Duke University, Durham, North Carolina; University of Virginia, Charlottesville, Virginia; National Institute of Health, Bethesda, Maryland; National Institute of Health, Bethesda, Maryland; Scripps Research, San Diego, California

## Abstract

**Background:**

Metformin has decreased SARS-CoV-2 RNA in 4 cell lines. In a RCT of > 1,000 majority vaccinated outpatients, COVID-19 related ED visits/Hospitalization/Death occurred in 4.7% of the metformin vs 9.0% of the placebo group by Day 28; clinician-diagnosed Long Covid (LC) occurred in 6.2% of metformin vs 10.3% of placebo by Day 300; and 14% of metformin vs 23% of the placebo group had detectable nasal viral load on Day 10. In a trial of 20 adults, 60% of metformin vs 100% of placebo had detectable viral load by Day 4. Observational analyses report associations between prevalent metformin and less severe acute COVID. Given these data, we assessed whether metformin currently prevents LC.

Cumulative Incidence Curve

**Figure:**
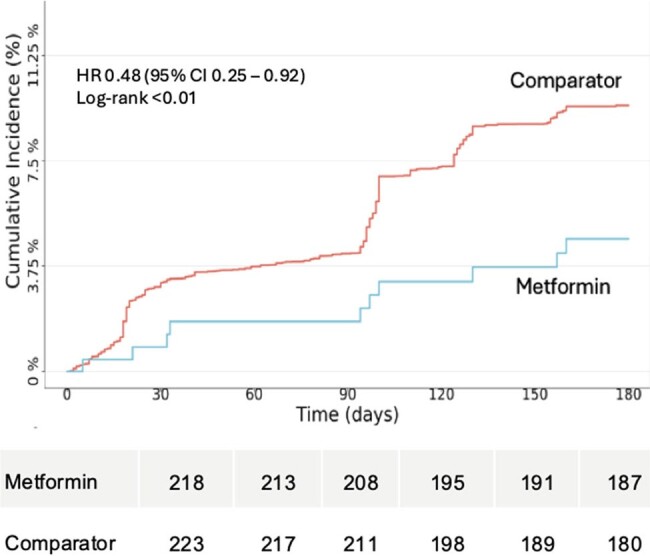

Cumulative incidence curves for the outcome of Long Covid/Death in the 180 Days after a metformin prescription (blue) or active comparator prescription (red). The increase in outcomes at Day 90 reflects the computable phenotype, which cannot be computed until 90 days after infection. Per data use requirements in the National Covid Cohort Collaborative (N3C), the starting number at risk is not shown due to small cell sizes or the ability to calculate small cell sizes.

**Methods:**

We emulated a randomized trial of metformin vs. control in SARS-CoV-2-infected outpatients. Intervention: prescription for metformin within 6 days of infection. Control: prescription for fluvoxamine, fluticasone, ivermectin, or montelukast (drugs used off-label for Covid but clinical trials have shown no effect on acute COVID outcomes). Exclusions: age < 18; metformin or comparator prescribed within 365 days; indication for chronic metformin use; contraindication for metformin or control. Outcome: a composite of LC or Death (LC/D), LC defined by U09.9 or a symptom/condition based computable phenotype. We used entropy balancing to estimate the average treatment effect with a weighted log linear model.

This is a forest plot showing metformin use versus active comparator use and the development of Long Covid/Death by Day 180.

**Figure:**
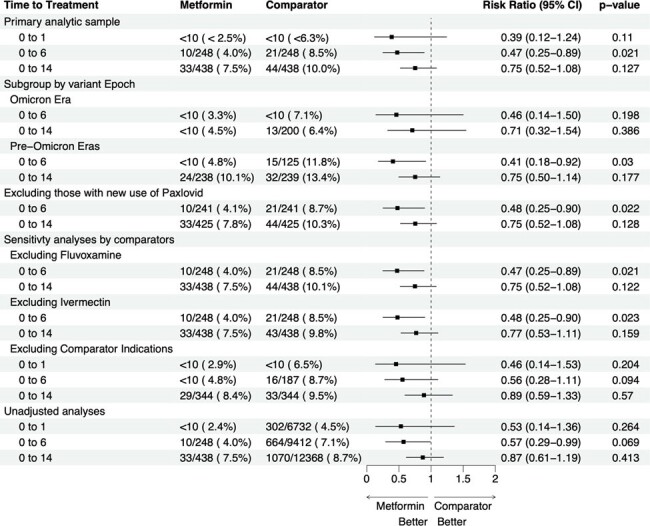

The black squares represent risk ratios (RR), and the lines represent 95% confidence intervals. The number of outcomes, denominators, and exact percentages are sometimes omitted so that cell sizes <10 are not able to be calculated, in accordance with the data use requirements in the National Covid Cohort Collaborative (N3C). The Pre-Omicron Era is larger than the Omicron era in both the Metformin and Comparator Cohorts, but the denominator for the Day 0-6 sample is not shown because of cell sizes <10. The subgroup of those with new Paxlovid use was too small to analyze, so we present only the subgroup who did not also receive a prescription for Paxlovid.

**Results:**

After balancing, the standardized mean differences were < 0.01. Overall, most (5,787/9,660, 59.9%) were infected during Omicron. In the metformin and control groups, 16% and 17% were Black; 16% and 13% were Hispanic, respectively. In the metformin group, 10/248 (4.0%) developed LC/D vs. 21/248 (8.5%) in the control group, RR 0.47 (95% CI 0.25 to 0.89). For prescriptions on Days 0-1 relative to infection the RR was 0.39 (95% CI 0.12-1.24); for prescriptions on Days 0-14 the RR was 0.75 (95% CI 0.52-1.08).

Cohort Balance

**Figure:**
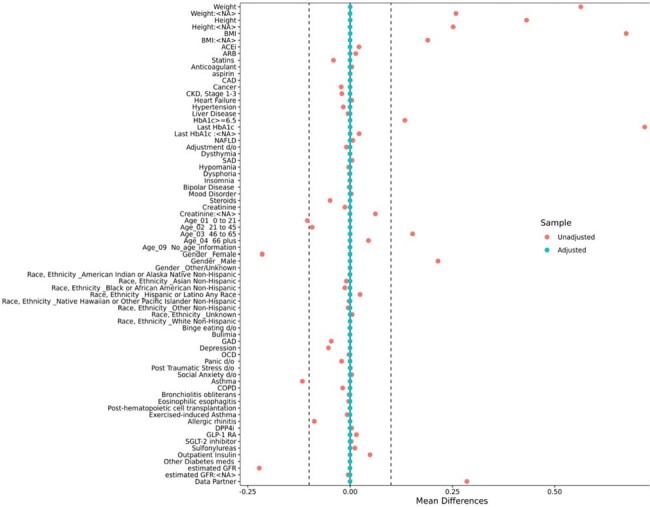

Cohort balance of the standardized mean differences (SMD) before

weighting (in pink), and after weighting (in teal), for the primary analysis.

**Conclusion:**

Metformin prescribed within a week of SARS-CoV-2 infection was associated with a 53% reduction in LC/D. Anything between an 11% to 75% reduction in risk is highly compatible with our data. Important questions remain: are results consistent in randomized clinical trial data from adults with a normal body mass index and those previously infected with SARS-CoV-2.

**Table 1:**
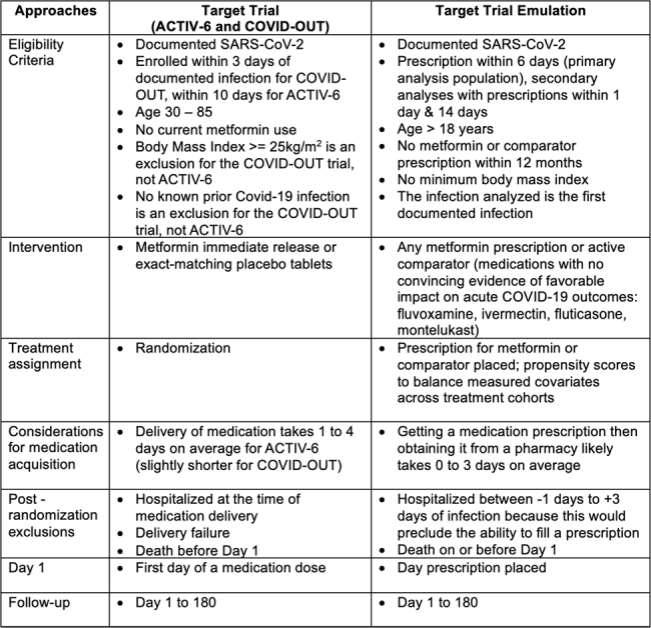
Individuals who experienced death before the prescription could be started were excluded from the trial emulation to mimic both target trials (COVID-OUT and ACTIV-6), as they were both decentralized trials that entailed medication delivery to the home. Those who experienced hospitalization between -1 to 3 Days were also excluded to mimic the target trial: those who were hospitalized on Day -1 to 0 relative to infection could not have received and taken an outpatient prescription of metformin or comparator for their SARS-CoV-2; those hospitalized on Days 1 to 2 likely spent at least one day in the emergency department before hospitalization, so it is also unlikely that they received an outpatient prescription, obtained the prescription from a pharmacy, and took a dose before being hospitalized.

**Disclosures:**

**All Authors**: No reported disclosures

